# The Kinetic Behaviors of H Impurities in the Li/Ta Bilayer: Application for the Accelerator-Based BNCT

**DOI:** 10.3390/nano9081107

**Published:** 2019-08-02

**Authors:** Xiao Liu, Huaican Chen, Jianfei Tong, Wenhao He, Xujing Li, Tianjiao Liang, Yuhong Li, Wen Yin

**Affiliations:** 1 School of Nuclear Science and Technology, Lanzhou University, Lanzhou 730000, China; 2 Institute of High Energy Physics, Chinese Academy of Science (CAS), Beijing 100049, China; 3 Spallation Neutron Source Science Center, Dongguan 523803, China

**Keywords:** BCC/BCC bilayer, H solution energy, electron density, diffusion barrier, hydrogen bubble

## Abstract

Hydrogen bubble phenomenon is one of the key issues to be solved in the development of a long-life target system for boron neutron capture therapy (BNCT). In this study, we assessed the kinetic behaviors of H impurities in the nano-composite target from the atomic level. Firstly, two kinds of Li/Ta nanolayer models were constructed, based on the calculated lattice parameters and surface energies. The H solution energy, diffusion mechanism, and hydrogen bubbles formation in the Li/Ta nanostructured bilayer were studied, through theoretical modeling and simulation. Our results show that the Li/Ta interfaces are effective sinks of H atoms because the H solution energies in the interface are lower. Meanwhile, due to the relatively low diffusion barriers, the large-scale H transport through the interface is possible. In addition, although it is more likely to form hydrogen bubbles in the Ta layer, compared with the Li layer, the anti-blistering ability of Ta is more impressive compared with most of other candidate materials. Therefore, the Ta layer is able to act as the hydrogen absorber in the Li/Ta bilayer, and relieve the hydrogen damage of the Li layer in the large-scale proton radiations.

## 1. Introduction

Malignant tumors remain a severe threat to human health [1]. In general, there are four major methods for malignant-tumor treatment: Surgery, radiation therapy, chemotherapy, and immunotherapy [2,3]. The accelerator-based boron neutron capture therapy (BNCT) is one of the most promising methods used to treat malignant tumors, due to its high treatment accuracy to the tumor cells and treatment ability to the deep-body tumors, as well as the advantages of low cost and easy maintenance, [4,5]. In recent decades, the accelerator-based BNCT attracted significant attention [4,5,6,7,8]. The accelerator-based BNCT has recently been developed in the China Spallation Neutron Source (CSNS). Based on the ^7^Li(p, n)^7^Be reaction, neutrons are generated from the bombardment of the Li target by 3.5 MeV protons [9,10].

The neutron-target system is recognized as one of the key factors in keeping the stability of the whole BNCT system [4,7]. Generally, for those neutron sources which, based on the ^7^Li(p, n)^7^Be reaction, the neutron-target system comprises the Li-film target and a metallic substrate (such as Cu or Al) for structural integrity and target-cooling [7,8]. However, high H concentration in the Li film can cause significant structural changes, such as cracks, surface roughening, and blistering, which can seriously degrade the heat conduction and service life of neutron-targets [4,5,11,12,13,14]. Hence, an innovative design was put forward to protect the target system from the deleterious effects to hydrogen, i.e., by adding a hydrogen-absorber nano-film, with a high H diffusion coefficient between the Li film and the Cu (or Al) substrate [8,14]. Because of the advantages of anti-blistering, few productions of the fast neutrons, and low dose-rate level of accompanying X-ray and gamma radiation, the Ta film is considered as the hydrogen absorber of the BNCT target system in CSNS and Budker Institute of Nuclear Physics [8,14].

At present, controlling the production, transport, and annihilation of hydrogen is the primary focus in developing the proton-irradiation-tolerance materials. For this purpose, the kinetic behaviors of H impurities in the interfaces and grain boundaries (GB) have been widely investigated [15,16,17,18,19,20,21]. For example, the Cu/Nb nano-layered composites possess significant radiation tolerances, due to the large amounts of defect-sinks of the interface [16]. The similar phenomenon was also observed in other interfaces and GBs [20,22]. Since the lattice mismatch is not as large as Kurdjumov-Sachs interfaces, the kinetic behaviors of impurity atoms in the BCC-BCC interfaces are always ignored, which results in little related investigations [23,24]. Therefore, much more efforts on H diffusion mechanisms and hydrogen bubble formation, in the BCC/BCC interfaces are urgently needed, in order to more accurately estimate the performance of the BCC/BCC bilayer in the radiation environment. To better understand these microcosmic behaviors, atomic-level modeling and simulation may provide powerful guidance and supplement experimental studies.

The first-principles calculation method based on the density functional theory (DFT) was employed in the present work. Two kinds of stable Li/Ta bilayer models were constructed based on the calculated lattice parameters and surface energy. Then the H solution energy, diffusion mechanism, and hydrogen bubbles formation in the bilayer were discussed in detail. Our results suggest that the Ta layer can act as the hydrogen absorber in the neutron-target system of BNCT and relieve the hydrogen damage of the Li target. In addition, the study methods and conclusions in the present paper are applicable to other BCC/BCC bilayers, which may be helpful in the design and screening of radiation-tolerant layered composites.

## 2. Materials and Methods 

The present first-principles calculation was performed through the pseudo-potential plane-wave method, which was implemented using the Vienna Ab initio Simulation Package (VASP) [25,26]. The generalized gradient approximation (GGA) with the Perdew-Burke-Ernzerhof (PBE) function was used to describe the exchange and correlation effects [27]. The structural relaxation of bulk Li and Ta converged at a 340 eV cutoff energy and a 5 × 5 × 5 k-point mesh. The 5 × 5 × 1 k-point meshes were employed for all the surface calculations, while it was set to 5 × 7 × 1 for both Li(100)/Ta(100) and Li(110)/Ta(110) bilayers. The energy minimization stopped when the forces on each atom less than 0.01 eV/Å. 

The surface energy ($\gamma_{s}$) is defined as the energy required (per unit area) to split the crystal into two separate parts along a specific plane. Usually, it can be defined as [28,29]:

(1) $$\gamma_{s}=\frac{E_{slab}-N\times E_{X}}{2\times A_{s}}$$

where $E_{slab}$ represents the total energy of the slab model, $E_{X}$ is the energy per atom of the bulk structures, N is the atom number of the slab model. $A_{s}$ represents the area of the surface. Factor 2 originates from two same surfaces in the slab model.

The work of separation ($W_{\mathrm{sep}}$) is the energy required every unit area when the bilayer is separated into two individual layers [28], it can be defined as:

(2) $$W_{\mathrm{sep}}=\frac{E_{\alpha }+E_{\beta }-E_{\alpha /\beta }}{A_{i}}$$

where $E_{\alpha /\beta }$ represents the total energy of the multilayer, $E_{\alpha }$ and $E_{\beta }$ are energies of the *α*, and *β* layers, respectively, $A_{i}$ represents the area of the interface.

The interface energy ($\gamma_{\mathrm{int}}$) is the excess energy (every unit area) due to the formation of the interface [28]:

(3) $$\gamma_{\mathrm{int}}=\frac{E_{\alpha /\beta }-M\times E_{\alpha }^{bulk}-N\times E_{\beta }^{bulk}}{A_{i}}-\gamma_{\alpha }-\gamma_{\beta }$$

where $E_{\alpha /\beta }$ represents the total energy of the *α*/*β* bilayer with *M* × *α* and *N* × *β* atoms, $\gamma_{\alpha }$ and $\gamma_{\beta }$ represent the surface energies of the *α*, and *β* layers, respectively.

The H solution energy at the interstitial site is defined as [30,31]:

(4) $$E_{\mathrm{int}}^{\mathrm{sol}}=E_{\left(\alpha /\beta ,1H\right)}-E_{\alpha /\beta }-\frac{1}{2}E_{H}{}_{{}_{2}}$$

where $E_{\left(\alpha /\beta ,1H\right)}$ represents the total energy of the bilayer containing an H atom.

The average H trapping energy at a vacancy can be defined as [30,31]:

(5) $$E_{\mathrm{vac}}^{trap}=\frac{1}{n}\left[E_{\left(\alpha /\beta ,1V,n\times H\right)}-E_{\left(\alpha /\beta ,1V\right)}\right]-\left[E_{\left(\alpha /\beta ,1H_{\mathrm{int}}\right)}-E_{\alpha /\beta }\right]$$

where $E_{\left(\alpha /\beta ,1V,n\times H\right)}$ represents the total energy of the *α*/*β* bilayer with *n* × H atoms in the vicinity of the vacancy, $E_{\left(\alpha /\beta ,1V\right)}$ is the total energy of the *α*/*β* bilayer containing a vacancy, $E_{\left(\alpha /\beta ,1H_{\mathrm{int}}\right)}$ is the total energy of the bilayer with an H atom in the interstitial site.

## 3. Results and Discussion

### 3.1. Bulk Properties and Surface Energy

Firstly, the lattice parameters and bulk modulus of the BCC-Li and BCC-Ta were calculated, and the results are listed in Table 1. The calculated results of the lattice parameters and bulk modules are in good agreement with previous studies [29,32,33,34,35,36], indicating that the structural models, used in the present study, are sufficiently reliable for use in further investigations.

The surface energies of three low-index surfaces for Li and Ta were calculated using the slab models with two free surfaces and a 16 Å vacuum-layer. To guarantee the thickness of the slab models are sufficed to exhibit the surface characteristics of the bulk materials, convergence tests were carried out, and seven atomic layers were finally applied in the present study [28]. As shown in Table 2, the (100) plane in Li and (110) plane in Ta possess the lowest surface energies, respectively, which is very consistent with previous studies [29,37,38,39,40]. It is also evidence of the accuracy and reliability of the calculation parameters and geometric models of the present paper.

### 3.2. The Interface Model Geometry

**Figure 1:**
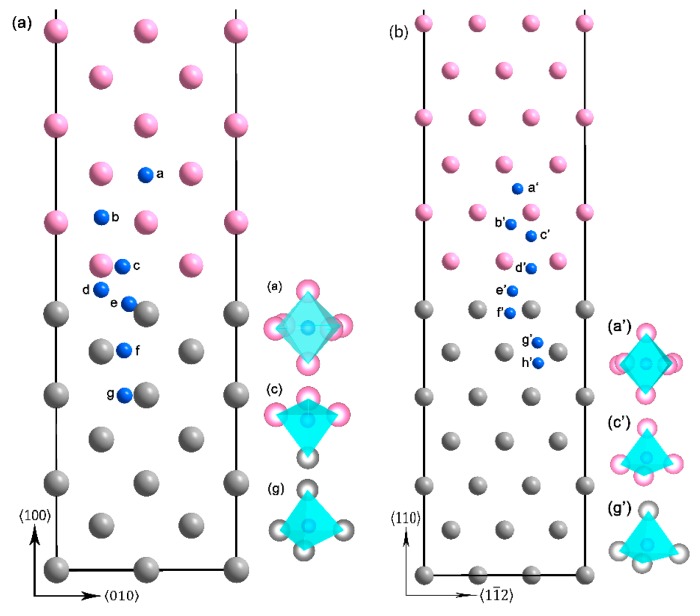
The geometric structures of (**a**) Li(100)/Ta(100) and (**b**) Li(110)/Ta(110) bilayers. The pink, gray, and blue atoms represent the Li, Ta, and H atoms (the H atoms here are used for the later calculation of the solution energy), respectively. The a-site and b-site in (**a**), and the a’-site in (**b**) are octahedral interstitial site (OIS), the rest sites are tetrahedral interstitial site (TIS).

Because the (100) plane in Li, and (110) planes in Ta, respectively possess the lowest surface energies. The Li(100)/Ta(100) and Li(110)/Ta(110) bilayers were constructed based on the calculated lattice parameters (see Figure 1). Actually, the Li(100)Ta/(110) bilayer was also considered. The disordered atomic configuration after the structural relaxation and the negative *W*_sep_ indicate that the structure model is energetically unfavorable. Hence, just the Li(100)/Ta(100) and Li(110)/Ta(110) bilayers were considered in the present paper. For the Li(100)/Ta(100) bilayer, the internal strains in [0 1 0] and [0 0 1] directions are both 1.896%, and for the Li(110)/Ta(110) bilayer, the internal strains in [$1\;\bar{1}\;2$] and [$1\;\bar{1}\;\bar{1}$] directions are 1.948%, and 1.916%, respectively.

**Figure 2:**
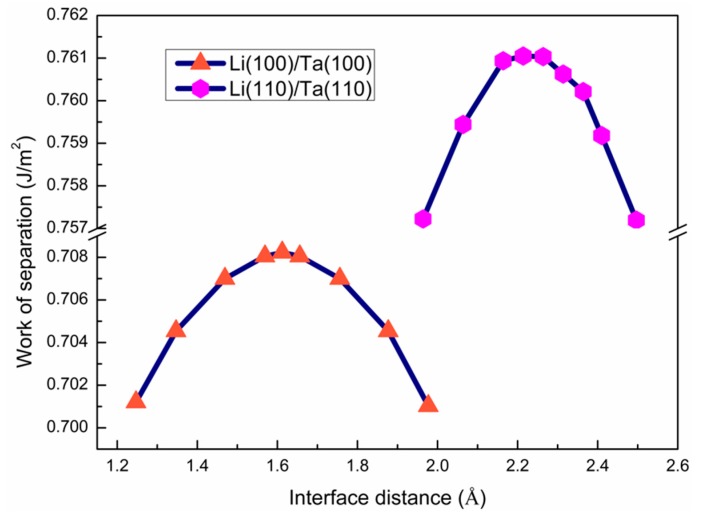
The *W*_sep_ as a function of the initial interface distance (d_0_) in both Li(100)/Ta(100) and Li(110)/Ta(110) bilayers.

To determine the initial interface distance (d_0_) of the bilayer models, *W*_sep_ as a function of d_0_, was calculated and plotted in Figure 2. The optimal d_0_ for the Li(100)/Ta(100) and Li(110)/Ta(110) bilayers are 1.612 Å, and 2.214 Å, respectively. The positive *W*_sep_ values mean that both interfaces are mechanically stable [28]. Meanwhile, the interface energy ($\gamma_{\mathrm{int}}$) was also calculated to evaluate the thermodynamic stability and nucleation resistance of both interfaces. The $\gamma_{\mathrm{int}}$ values for the Li(100)/Ta(100) and Li(110)/Ta(110) interfaces are 1.606, and 1.245 eV, respectively. Positive $\gamma_{\mathrm{int}}$ values mean that both interfaces are thermodynamically stable [28]. Therefore, both Li(100)/Ta(100) and Li(110)/Ta(110) interfaces are mechanically and thermodynamically stable.

### 3.3. The H Solution Energy and Diffusion Mechanism

**Figure 3:**
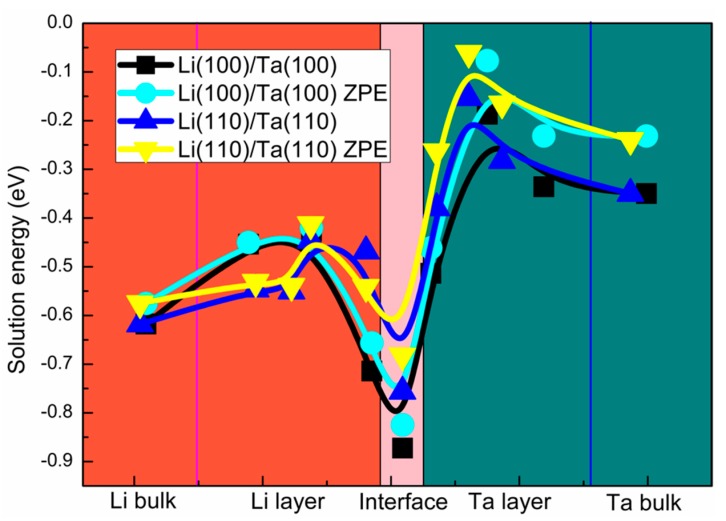
The solution energies of the H atoms at the interstitial sites in Li(100)/Ta(100) and Li(110)/Ta(110) bilayers.

The nucleation and growth of macroscopic hydrogen bubbles originate from H diffusion, which pertains to the large-scale jumps of the H atoms among the interstitial sites [41,42,43]. Therefore, the solution energies of the H atoms, at the interstitial sites, were calculated. Two kinds of interstitial sites, including the tetrahedral interstitial site (TIS) and the octahedral interstitial site (OIS), were considered in the present study. In the bulk Li, the OIS is energetically favorable with the $E_{\mathrm{int}}^{\mathrm{sol}}$ of −0.617 eV (0.180 eV lower than that in the TIS). While in the bulk Ta, the H atoms prefer the TIS with the $E_{\mathrm{int}}^{\mathrm{sol}}$ of −0.350 eV (0.179 eV lower than that in the OIS), which is consistent with the previous BCC metals [44,45,46,47]. The similar phenomenon is also found in the Li/Ta bilayer, as shown in Figure 1, the H atoms are stable at the OIS in those Li layers away from the interface, while the H atoms prefer the TIS in both, Ta layers and those Li layers close to the interface. The variation trend of $E_{\mathrm{int}}^{\mathrm{sol}}$ along the vertical direction to the interface is displayed in Figure 3. The negative $E_{\mathrm{int}}^{\mathrm{sol}}$ in both Li and Ta layers indicates that both layers have a relatively high H-storage capacity. Meanwhile, $E_{\mathrm{int}}^{\mathrm{sol}}$ in the Li layers are obviously lower than those in the Ta layers, which indicates that the H-storage capacity of the Li layer is higher than that of the Ta layer. In addition, a drastic drop of $E_{\mathrm{int}}^{\mathrm{sol}}$ can be found around the interface, indicating that the H-rich layer may be formed in the interface in the proton radiation environment. The Zero Point Energy (ZPE) corrections, for the H and Li atoms in this study, are considered due to their lighter mass. The ZPE energy was calculated from the vibrational frequencies: $E_{ZPE}=\frac{1}{2}\sum h\nu$, where ʋ is the real frequency. The ZPE corrections of the Li(100)/Ta(100) bilayer are in the range of 0.84~0.89 (H in the Li layers) and 0.89~0.94 eV (H in the Ta layers), respectively. And those of the Li(110)/Ta(110) bilayer are in the range of 1.12~1.14 (H in the Li layers), and 1.20~1.22 eV (H in the Ta layers), respectively. The ZPE energies of the ideal Li(100)/Ta(100) and Li(110)/Ta(110) bilayer are 0.70, and 0.97 eV, respectively. Therefore, the corrected H solution energies were obtained and displayed in Figure 3. Although the ZPE corrections lead to a slight increase in H solution energy, the variation trend remains unchanged. And what should be noted is that the corrections for the H atoms in the Ta layers seem more pronounced.

**Figure 4:**
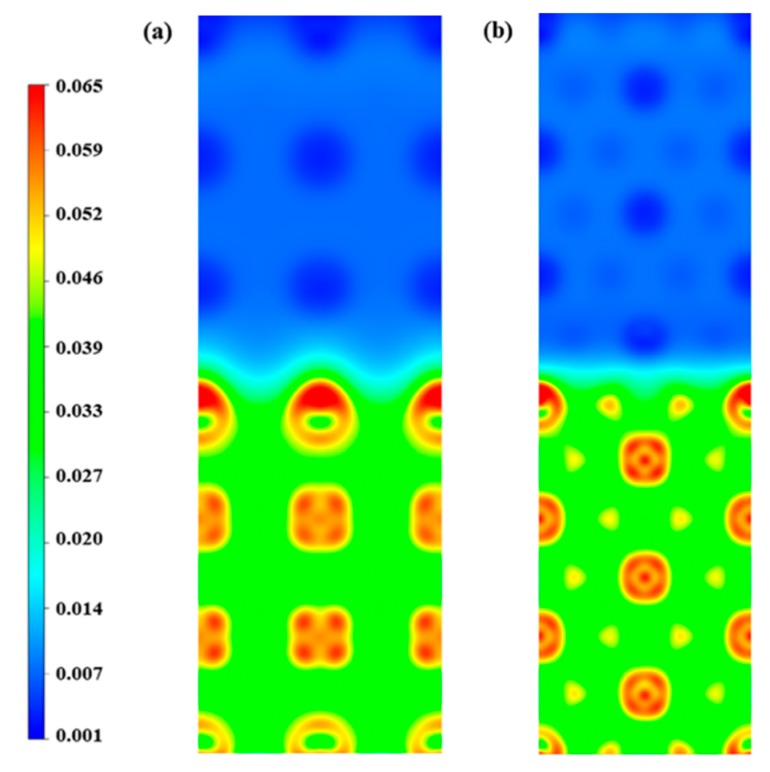
The charge densities of (**a**) Li(100)/Ta(100) and (**b**) Li(110)/Ta(110) bilayers (in e/Bohr^3^). The upper and lower half-part is the Li, and Ta layer, respectively.

**Figure 5:**
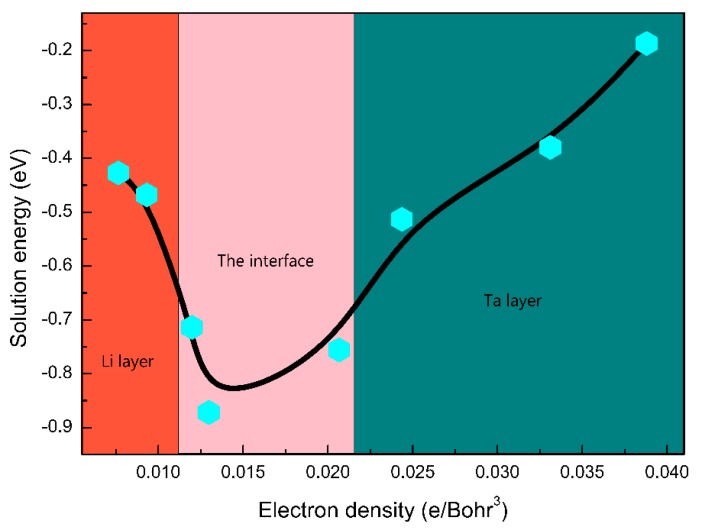
The solution energy of the H atoms as a function of the electron density in the Li/Ta bilayer (the ZPE correction is not considered here).

From a physical standpoint, the solution energy of the H atoms in metals can be interpreted according to the homogeneous electron gas theory [48,49]. The charge density of the Li/Ta bilayer is shown in Figure 4. The charge density of the Li layer is obviously lower than that of the Ta layer. And the charge density of the interface falls in between the Li and Ta layers. Based on the electron density of Li(100)/Ta(100) and Li(110)/Ta(110) bilayers, the specific correlation between the H solution energy and the electron density is displayed in Figure 5. The solution energy of the H atoms decreases monotonously with the increasing electron density, achieving the minimum at the critical density (approximately 0.013 e/Bohr^3^), and then increasing monotonously with the increase in electron density, as consistent with previous studies [30,31,48,49]. The critical electron density appeared in the Li side of the interface, as a result, $E_{\mathrm{int}}^{\mathrm{sol}}$ in the interface is the lowest. 

**Figure 6:**
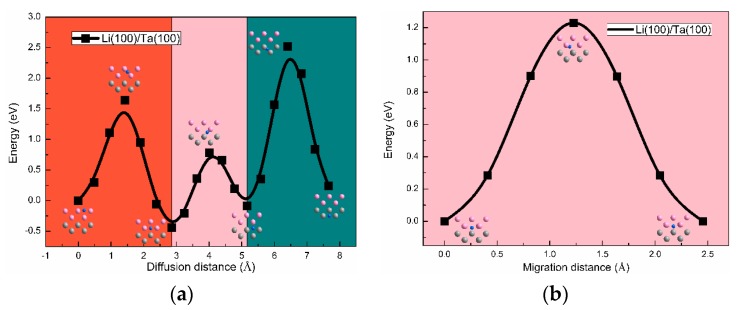

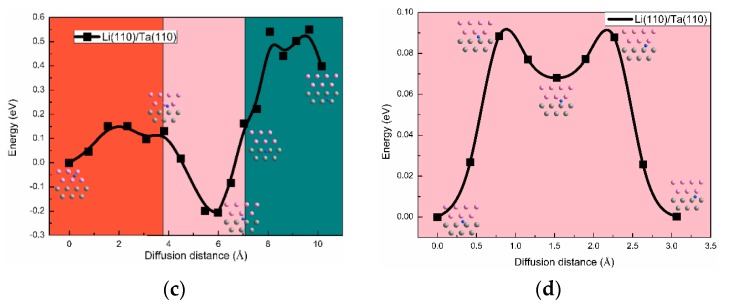
The diffusion barriers for the H atoms migrate through (**a**) and along (**b**) the Li (100)/Ta(100) interface. The diffusion barriers for the H atoms migrate through (**c**) and along (**d**) the Li (110)/Ta(110) interface.

Diffusion barrier calculations, based on the climbing image nudged elastic band (CI-NEB) method, were performed in order to clarify the H diffusion mechanism in Li/Ta bilayer [50]. Two kinds of diffusion behaviors for the H atoms, including diffusing through the interface and diffusing in the interface, were considered for both Li(100)/Ta(100) and Li(110)/Ta(110) bilayers. As shown in Figure 6, the diffusion barriers in Li(110)/Ta(110) are much lower than those in Li(100)/Ta(100) bilayer, showing that the H diffusion behaviors are strongly related to the crystal orientation. In the Li(110)/Ta(110) bilayer, the diffusion barrier is approximately 0.15 eV when the H atoms diffuse from Li or Ta layer to the interface. Because of the lowest $E_{\mathrm{int}}^{\mathrm{sol}}$ in the Li/Ta interface and the relatively low diffusion barriers, it can be predicted that the diffusion of the H atoms from Li or Ta layer toward the interface can easily take place. That, is to say, the interface has the ability to absorb the H atoms. When the H atoms are in the process of diffusing in the interface, the barrier is calculated at approximately 0.09 eV. Such a small barrier enables the large-scale H diffusion in the interface and the formation of an H-rich layer at the interface. In addition, the diffusion barriers of the H atoms, from the interface to the Li and Ta layer, are approximately 0.35, and 0.75 eV, respectively. Considering the barrier is not large enough, the H atoms diffusing through the interface is possible, and the diffusion direction depends on the differences in the H concentration of each side.

### 3.4. The H Bubbles Formation in the Li/Ta Bilayer

**Figure 7:**
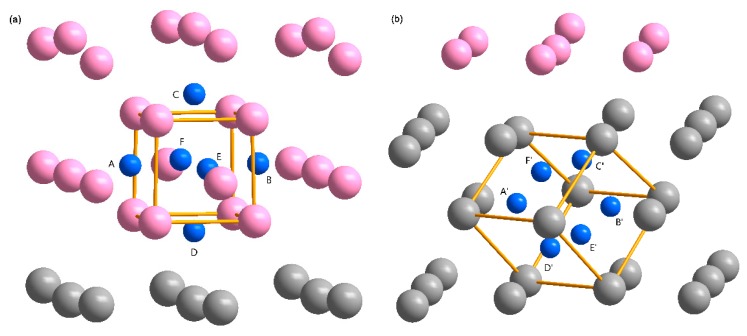
The atomic configurations of the H clusters in (**a**) Li(100)/Ta(100) and (**b**) Li(110)/Ta(110) bilayers. The atomic configurations of the H clusters in the Li and Ta layers are mirror images of each other, therefore, the configurations in the Li layer of the Li(100)/Ta(100) bilayer and that in the Ta layer of the Li(110)/Ta(110) bilayer are displayed here for a brief description. The pink, gray, and blue atoms represent the Li, Ta, and H atoms, respectively. The hollow box represents a vacancy.

Generally, the H atoms and vacancies are strongly attractive mutually in metals [30,31,46]. According to the aforementioned homogeneous electron gas discussions (see Section 3.3), it is reasonable to attribute this strong attraction to the low electron density in the vicinity of the vacancies. Therefore, the pre-existing vacancies can act as H trapping sites, where hydrogen bubbles nucleate and grow. According to the vacancy trapping mechanism of hydrogen bubbles [30], the formation of the vacancy leads to the reduction of the local charge density, providing a spherical charge iso-surface to accommodate the H atoms. On the other hand, the gathering of H atoms leads to a local strain field, which is the origin of the lattice distortion and expansion. Accordingly, the electron density is altered, thereby, making it more energetically favorable to trap even more H atoms in the vicinity [44,47]. Thus, hydrogen bubbles nucleate and grow in the vacancy once the H density reaches a critical value. According to the above-mentioned nucleation mechanisms of hydrogen bubble, H atoms prefer to occupy those sites with the same charge density on the spherical charge iso-surface around the vacancy, which is in accordance with the aforementioned homogeneous electron gas discussions (see Section 3.3). In addition, the previous study has shown that a mono-vacancy in the bulk-Ta can hold up to 6 H atoms [46]. Therefore, six H atoms around the vacancy are sufficient to illustrate the differences of the hydrogen bubbles formation in the Li and Ta layers (see Figure 7). 

To study the formation of hydrogen bubbles in the Li/Ta bilayer, the average H-trapping energy, in two kinds of bilayer models, was calculated. In the Li(100)/Ta(100) bilayer, three kinds of H clusters were taken into consideration. One is the two-H cluster (configurations: A-B and C-D), another one is the four-H cluster (configurations: A-B-C-D and C-D-E-F), and the third one is the six-H cluster (configuration: A-B-C-D-E-F), as shown in Figure 7a. Configurations of C-D and C-D-E-F are more energetically favorable for the two-H and four-H clusters in both Li and Ta layers (approximately a few tenths of eV lower than the other one). In the Li(110)/Ta(110) bilayer, three kinds of H clusters were taken into consideration. One is the two-H cluster (configurations: A’-B’ and C’-D’), another is the four-H cluster (configuration: A’-B’-C’-D’), and the third one is the six-H cluster (A’-B’-C’-D’-E’-F’), as shown in Figure 7b). Configuration of C’-D’ is more stable for the two-H cluster in both Li and Ta layers.

**Figure 8:**
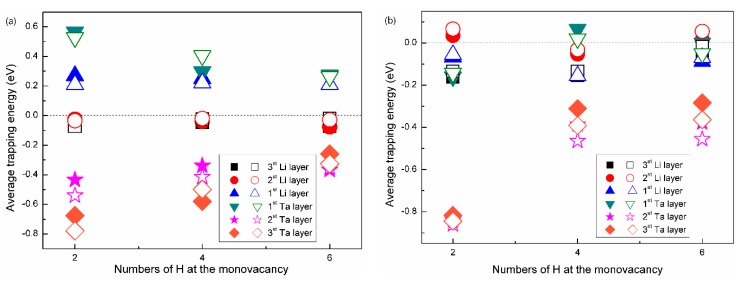
The average H-trapping energy as a function of numbers of the H atoms in the mono-vacancy in (**a**) Li(100)/Ta(100) and (**b**) Li(110)/Ta(110) bilayers. The hollow patterns represent the trapping energies with the ZPE corrections, while those solid ones not. According to the definition of the H trapping energy, the interstitial H atoms for reference can lie at the first, second and third layers (the nearest layer to the interface is recognized as the first layer, and so on). The vacancy was set on the second Li or Ta layers.

The average H-trapping energy, as a function of the H numbers in a mono-vacancy, was plotted in Figure 8. $E_{\mathrm{vac}}^{trap}$ can be understood as the energy variation when the H atoms migrates from the interstitial sites to vacancies, and the negative value means a exothermic process [30,31]. Therefore, considering the different reference states for the H atoms, those H atoms which tend to be bound to vacancies can be distinguished. As shown in Figure 8a,b, the variation trends of the average H-trapping energy for both Li(100)/Ta(100) and Li(110)/Ta(110) bilayers are similar, no matter whether ZPE correction was performed or not. Taking the Li(100)/Ta(100) bilayer for example, when the interstitial-H is located in the first Li (or Ta) layer, positive $E_{\mathrm{vac}}^{trap}$ indicates that it is difficult for a vacancy to trap the H atoms near the interface. Meanwhile, $E_{\mathrm{vac}}^{trap}$ stays negative when the interstitial-H is located in the second and third Li (or Ta) layers, which indicates that the H-trapping direction is in accordance with the diffusion directions of the H atoms.

As shown in Figure 8b, $E_{\mathrm{vac}}^{trap}$ increases with increasing H numbers inside the vacancy, which is in accordance with the variation trend of Ta, V, Nb, Cr, Mo, and W [30,31,46]. Physically, the sinking of H atoms leads to an alteration of the local strain and charge fields [47,51]. At the initial stage of nucleation and growth of the H bubble, H density is relatively low, while, the local strain and charge fields are close to spherical. The average H-trapping energy is negative, so it is energetically favorable for other H atoms to join the H bubble at this stage. The H density continuously increases as the bubble grows, until the local strain and charge fields are no longer spherical, then the average H-trapping energy becomes positive, so it is energetically unfavorable for the growth of H bubble at this stage. In addition, it is worth noting that $E_{\mathrm{vac}}^{trap}$ fluctuates around the reference line in the Li layer, indicating that it is not likely to form the large-scale hydrogen bubbles in the Li layer. Although the trapping energy in the Ta layer remains negative, the $E_{\mathrm{vac}}^{trap}$ for the six-H cluster is about −0.40 eV, obviously larger than that in the bulk-Mo (−0.85 eV) or bulk-W (−1.05 eV) [30,31]. Meanwhile, the trapping energy of bulk Ta proved to be clearly larger than those in the bulk Cr, Mo, and W [46]. Therefore, the Ta layer is supposed to possess an impressive anti-blistering ability. Actually, large numbers of protons were implanted in the Li layer in BNCT, resulting in a higher H concentration in the Li layer of the Li/Ta bilayer. Hence, the H atoms in Li layer migrate toward the Li/Ta interface, which significantly impedes the formation of hydrogen bubbles in the Li film. When the H concentration in the interface reaches saturation, on the account of the large differences of the H concentration between the Li and Ta layers, the H atoms can then overcome the barrier and diffuse from Li to Ta film, which causes a large-scale H transport. Meanwhile, although it is more likely to for the H bubbles in the Ta layer to form compared with the Li layer, its anti-blistering ability is more impressive compared with most of the candidate materials [14,46]. Therefore, the Ta layer is believed to be the proper hydrogen absorber in the target system of BNCT.

## 4. Conclusions

In conclusion, the numerical calculations, based on DFT, were performed, in order to study the geometrical structure, H diffusion mechanism, and hydrogen bubbles formations in the Li/Ta nanostructured bilayer. The work of separation and interface energy indicate that both Li(100)/Ta(100) and Li(110)/Ta(110) interfaces are mechanically and thermodynamically stable. Based on the H solution energy and the electron density, we can conclude that the Li/Ta interface possess a good ability to conduct H sinking, due to the optimal electron density at the interface. Meanwhile, the H atoms can diffuse through the interface, due to the relatively lower diffusion barriers. Hence, the large-scale H transport can take place in the Li/Ta bilayer. Additionally, the H-trapping energy of Ta layer is lower than that of Li layer, and higher than that of other candidate materials (Mo, W and V), which suggests that the Ta layer is a better hydrogen absorber. Due to the above-mentioned advantages, such as the H sinking ability of the interface, low barriers of the across-interface diffusion, and the higher H-trapping energy of the Ta layer, the Li/Ta nano-composite target is believed to possess an improved anti-blistering ability in the radiative application environment of BNCT.

## Figures and Tables

**Table 1:** The optimized lattice parameters and bulk modulus for the BCC-Li and BCC-Ta.

	a (Å)	Bulk Modulus (Mbar)	References (Å and Mbar)
Li	3.439	1.373	3.439 ^a^; 3.436, 1.406 ^b^; 3.479 ^c^
Ta	3.308	1.995	3.311, 1.96 ^d^; 3.309, 2.11 ^e^; 3.304, 1.947 ^f^

^a^ DFT calculations. [32]; ^b^ DFT calculations. [33]; ^c^ Experiments. [34]; ^d^ DFT calculations. [29]; ^e^ DFT calculations. [35]; ^f^ Experiments. [36].

**Table 2:** The calculated surface energies of BCC-Li and BCC-Ta (in J/m^2^).

	(100)	(110)	(111)
**Li**	0.456	0.500	0.522
**Ref.**	0.48 ^a^, 0.522 ^b^	0.51^a^, 0.556 ^b^,0.522 ^c^	0.56 ^a^, 0.590 ^b^
**Ta**	2.486	2.303	2.749
**Ref.**	2.32 ^d^	2.31, 2.90 ^e^	2.71

^a^ DFT. [37]; ^b^ Full charge density (FCD)-LMTO. [38]; ^c^ Experiments. [39]; ^d^ DFT. [29]; ^e^ Experiments. [40].
